# Patient characteristics, treatment patterns and clinical response in patients with treatment resistant depression starting a new treatment in the clinical setting: a multicenter, observational study in Portugal

**DOI:** 10.3389/fpsyt.2026.1749006

**Published:** 2026-08-13

**Authors:** Ricardo Moreira, Patrícia Frade, Nuno Campeão, Sofia Gomes, Alice Lopes, Georgina Lapa, João Vian, Ana Fonseca, Flávia Polido, Bernardo Barata, David Mota, Patrício Ferreira, Yaroslava Martins, Miguel Nascimento, Licínio Santos, João Mendes, Diana Durães, Luís Ferreira, Dulce Maia, Nuno Cunha, Bárbara Cochicho, Gonçalo Amorim, André Ponte

**Affiliations:** 1 Unidade Local de Saúde de São João, Porto, Portugal; 2 Unidade Local de Saúde do Oeste, Caldas da Rainha, Portugal; 3 Unidade Local de Saúde da Póvoa de Varzim/Vila do Conde, Vila do Conde, Portugal; 4 Unidade Local de Saúde de Santo António, Porto, Portugal; 5 Unidade Local de Saúde de Gaia/Espinho, Vila Nova de Gaia, Portugal; 6 Unidade Local de Saúde de Lisboa Ocidental, Lisbon, Portugal; 7 Unidade Local de Saúde da Região de Leiria, Leiria, Portugal; 8 Unidade Local de Saúde do Algarve, Faro, Portugal; 9 Unidade Local de Saúde do Arco Ribeirinho, Lisbon, Portugal; 10 Unidade Local de Saúde de Coimbra, Coimbra, Portugal; 11 Unidade Local de Saúde do Alto Minho, Viana do Castelo, Portugal; 12 Unidade Local de Saúde do Baixo Alentejo, Beja, Portugal; 13 Unidade Local de Saúde de São José, Lisbon, Portugal; 14 Hospital Dr. Nélio Mendonça, Funchal, Portugal; 15 Unidade Local de Saúde da Guarda, Guarda, Portugal; 16 Unidade Local de Saúde da Arrábida, Setúbal, Portugal; 17 Unidade Local de Saúde do Médio Tejo, Abrantes, Portugal; 18 Unidade Local de Saúde de Trás-os-Montes e Alto Douro, Vila Real, Portugal; 19 Unidade Local de Saúde de Viseu Dão-Lafões, Viseu, Portugal; 20 Johnson & Johnson Innovative Medicine, Lisbon, Portugal; 21 Hospital do Divino Espírito Santo, Ponta Delgada, Portugal

**Keywords:** clinical outcomes, depression, patient characterization, TRD, treatment

## Abstract

**Background:**

Treatment resistant depression (TRD) is a burdensome condition with a severe impact on patients’ everyday lives. Therapeutics have improved in the last decade, however, outcomes remain poor. Real-world characterization has become increasingly important to accurately depict the clinical setting. ResisToday was an observational, prospective, multicenter study that aimed to increase available real-world evidence by evaluating clinical and socio-demographics, treatment patterns and clinical and patient-reported outcomes (PROs) of patients with TRD in Portugal.

**Methods:**

Patients with a clinical diagnosis of TRD and moderate to severe depression (Montgomery-Åsberg Depression Rating Scale [MADRS] score ≥22), who were initiating a new treatment for TRD could enroll in ResisToday. Clinical outcomes were evaluated using the MADRS and the Clinical Global Impression-Severity scale (CGI-S). PROs were reported using the Patient Health Questionnaire-9 (PHQ-9) and EuroQol 5-Dimension 5-Level (EQ-5D-5L) questionnaire. Outcomes were assessed at baseline, Week 4 (primary endpoint) and Week 20.

**Results:**

Of 153 patients, 123 (80.4%) were female and the mean age was 53.9 years. The mean age of onset of clinical depression symptoms was 38.0 years, and the mean time since diagnosis of major depressive disorder (MDD) was 11.8 years. TRD treatments initiated at baseline were varied; the most common treatments initiated or ongoing at baseline were mirtazapine (43.1%), venlafaxine (42.5%), quetiapine (25.5%), and trazodone (20.9%). Most patients’ antidepressant treatment was augmented with an antipsychotic (36.6%), combined with additional antidepressants (22.2%), or combined with another augmentation strategy (19.0%). Improvements in MADRS were observed from baseline to Week 4 and Week 20 (both p<0.001). Clinical response and remission, assessed by MADRS, were achieved by 14.9% and 3.1% of patients, respectively, at Week 4, and 40.5% and 25.3% at Week 20. Improvements were observed across CGI-S, PHQ-9, and EQ-5D-5L measures through Week 20 (all p<0.001).

**Conclusions:**

Outcomes for patients with TRD in real-world clinical practice remain poor, with most patients not responding after 20 weeks of treatment. Treatment heterogeneity highlights a lack of clinical consensus, and the need for novel therapeutics and improved treatment protocols for TRD in real-world clinical practice in Portugal.

## Introduction

Depression is a leading cause of disability worldwide affecting around 5% of the global adult population (1). In particular, major depressive disorder (MDD) was ranked third globally in terms of disease burden in 2018 and is projected to be first by 2030 (2). MDD requires complex clinical management to address a broad spectrum of symptoms, such as fatigue, weight loss, low mood, and suicidality, and can impose a substantial negative impact on patients’ physical and mental health (2).

Treatment resistant depression (TRD) affects 10–30% of patients with MDD and can be defined as a failure to respond to two or more antidepressants at therapeutic doses, over an appropriate period of time, within the current depressive episode (3–7). TRD diagnosis and treatment can be difficult due to a lack of consensus regarding the definition of TRD and no standardized treatment pathway, with clinicians administering a wide variety of therapeutic strategies (6, 8). Furthermore, evidence suggests patients with TRD experience increased disease burden compared to their treatment-responsive counterparts, placing additional strain on clinical management (5, 9–13).

Psychopharmacological therapeutics, electroconvulsive therapy (ECT), as well as other non-pharmacological treatments that include traditional psychotherapy and transcranial magnetic stimulation (TMS), have shown some potential in treating TRD (14–18). However, despite the broad range of therapeutic options currently available for TRD, novel therapies that can provide a more effective and rapid response for patients are needed (19).

The use of RWE is valuable for assessing treatment efficacy across diverse patient populations in everyday clinical settings. For instance, patients with comorbidities and those receiving multiple medications are included in RWE studies, representing subgroups of patients who are often excluded from clinical trials to ensure internal validity. The inclusion of a broader range of patients with TRD allows RWE studies to offer insights that extend beyond clinical trial data by assessing a broader and more representative population of patients with TRD. Previous RWE studies have assessed treatment patterns, clinical and patient-reported outcomes (PROs), as well as patient characteristics in European patients with TRD (20–24). However, despite Portugal having one of the highest prevalence rates of depression in Europe, there is a lack of RWE and clinical guidance for the treatment of patients with TRD in Portugal (19, 25, 26).

As such, the ResisToday study aimed to increase the available Portugal-specific RWE by evaluating clinical and health-related quality of life (HRQoL) responses of patients with TRD starting a new treatment in clinical practice across Portugal. Additionally, the study aimed to characterize clinical and sociodemographic profiles and treatment patterns among these patients.

## Methods

### Study design and participants

ResisToday was an observational, prospective, multicenter, non-interventional study evaluating clinical response, PROs, treatment patterns and characterizing the clinical and sociodemographic profile of adults who started a new pharmacological or non-pharmacological treatment for TRD in a clinical practice setting in Portugal.

Patients were enrolled using a consecutive sampling strategy and all patients at participating sites who fulfilled the eligibility criteria were invited to participate when attending their regular consultation appointments. Included patients met the criteria for diagnosis of TRD, defined as lack of clinically meaningful improvement with two or more different oral antidepressants, given at an adequate dose and duration in the same major depressive episode, as determined using the Massachusetts General Hospital-Antidepressant Treatment Response Questionnaire (MGH-ATRQ) (23). A Montgomery-Åsberg Depression Rating Scale (MADRS) score of ≥22, denoting moderate or severe depression, was also required for inclusion; moderate and severe depression were defined as a MADRS score of 20–34 and >34, respectively. Patients with a current or prior diagnosis of a psychotic disorder or MDD with psychotic features, according to the Diagnostic and Statistical Manual of Mental Disorders Fifth Edition (DSM-5), bipolar or related disorders, obsessive compulsive disorder, intellectual disability, autism spectrum disorder, borderline personality disorder, antisocial personality disorder, histrionic personality disorder or narcissistic personality disorder were excluded (**Supplementary Table 1**). All patients had initiated a new antidepressant treatment to treat the current depressive episode. In the context of this study, a new antidepressant treatment was considered any new pharmacological or non-pharmacological treatment prescribed either to replace, or in addition to, the current antidepressant treatment. This did not include a dose increase of a current antidepressant treatment.

All patients provided written informed consent at the start of the study; baseline was defined as the date each patient signed the informed consent form. Data were collected in an electronic Case Report Form (eCRF) at baseline, 4-week (up to a maximum of 5 weeks), and 20-week (± 4 weeks) follow-ups. The end of study was defined as the last patient’s final visit, whether the patient completed the study or withdrew before Week 20. Patients who changed treatment during the study period were discontinued from the study following a final evaluation; patients who changed treatment prior to the Week 4 follow-up were considered non-responders for whom only safety and characterization information were collected.

### Outcomes

Demographic data were collected at baseline, including age, sex, educational level, and employment status. Clinical characteristics and disease history data were also collected, including age at diagnosis of MDD, number of depressive episodes, duration of the current episode, MADRS score, current medication, number of drugs failed in the current episode, and depression severity (assessed by MADRS score and Clinical Global Impression-Severity scale [CGI-S] score).

The primary objective was to evaluate clinical response, defined as ≥50% improvement in the MADRS total score from baseline to Week 4. Clinical response, remission (defined as MADRS total score of ≤10) and change in disease severity (assessed by CGI-S score) were also evaluated through Week 20. PROs, including the Patient Health Questionnaire-9 (PHQ-9), the EuroQol 5-Dimension 5-Level (EQ-5D-5L) questionnaire, and MGH-ATRQ, were also assessed through Week 20.

The MADRS scale is used to measure the severity of depressive episodes in patients with mood disorders. MADRS total score ranges from 0 to 60, with higher values indicating more severe depression (27). The CGI-S scale offers a clinician-determined assessment of overall disease severity; scores range from 0 to 7, with a score of 7 indicating that the patient is extremely ill (28). The PHQ-9 is used to assess the level of depression; total score for the nine items ranges from 0 to 27, with higher values denoting higher levels of depression (29). The EQ-5D-5L questionnaire is utilized to assess quality of life (30). It comprises five dimensions: mobility, self-care, usual activities, pain/discomfort, and anxiety/depression (30). Additionally, the EQ-5D-5L questionnaire includes the EQ visual analog scale (VAS), which ranges from 0 (worst health you can imagine) to 100 (best health you can imagine) (30).

### Statistical analysis

The sample size was calculated considering the primary objective to describe treatment clinical response at 4 weeks. As the study did not test a formal statistical hypothesis, a sample size of 150 patients was used, based on the expected number of eligible patients in the identified 27 participating sites over 18 months. All statistical analyses were performed based on the full analysis set (FAS), which comprised only patients that fulfilled the inclusion criteria.

Absolute and relative frequencies of patients with clinical and PRO responses are presented. Percent change in MADRS score and CGI-S score were assessed using the Wilcoxon test. Baseline MADRS score, age, gender, employment/occupational status, and duration of the current major depressive episode were selected as covariates for the generalized estimating equation (GEE) model. These characteristics were chosen as they represent important demographic and clinical factors in the treatment of TRD, aligning with the wider literature that identifies these characteristics as predictors of response or risk factors for TRD (age, gender, unemployment and severity (31); duration of current episode (32)). Models were adjusted for the score at baseline, age groups, gender, employment/occupational status and the duration of the current major depressive episode. The linear regression coefficient (B) and 95% confidence intervals (CIs) were estimated.

All statistical tests were two-sided with statistical significance set at p<0.05. All p values are nominal and not adjusted for multiplicity. Missing data were not imputed; all outcomes are reported as observed case.

## Results

### Patient disposition and baseline characteristics

Patient disposition is presented in **Supplementary Figure 1**. Of 174 patients enrolled in the study, 153 were included in the FAS. All patients included in the FAS completed the baseline visit, 131 patients completed Week 4, and 79 patients completed Week 20. Overall, 68 (44.4%) patients discontinued during the study period, of which 61.8% discontinued due to change or interruption in the current therapy (35.3% not related to adverse events [AEs]; 26.5% due to an AE). Other reasons for discontinuation included lost to follow up (22.1%), “other” (8.8%), new antidepressant prescribed but not initiated (2.9%), death (n=2 [2.9%]; neither of which were related to TRD treatment) and withdrawal of consent (1.5%).

Patient baseline demographics are presented in **Table 1**. At baseline, the patients’ mean age was 53.9 years, most patients were female (80.4%), and psychiatric disease was the most common factor impacting employment status (99/112; 88.4%). Most patients (53/88; 60.2%) in employment had lost working days related to psychiatric disease or its treatment during the last 12 months.

**Table 1:** Patient baseline demographics.

	Total (N = 153)
Age (years), mean (SD)	53.9 (10.6)
Sex, female, n (%)	123 (80.4)
Marital status, n (%)	
Single	9 (5.9)
Married	84 (54.9)
Divorced	32 (20.9)
Widowed	10 (6.5)
Civil union	14 (9.2)
Separated	4 (2.6)
Living status, n (%)	
At home, alone	18 (11.8)
At home, with family or friends	135 (88.2)
Employment/occupational status, n (%)	
Employed^a^	89 (58.2)
Unemployed^b^	28 (18.3)
Dependent on partner/spouse	3 (2.0)
Student	2 (1.3)
Retired	31 (20.3)
Employment/occupational status was impacted due to any reason, n (%)	N=112
Psychiatric disease	99 (88.4)
Other disease	7 (6.3)
Other reason	6 (5.4)
Not applicable (not impacted)	40
If employed, were any working days lost related to the disease and/or its treatment during the last 12 months? n (%)	N=88
Yes	53 (60.2)
If yes, number of working days lost in the last 12 months	N=52
Mean (SD)	158.6 (86.4)
Formal education, n (%)	N=150
Elementary school	66 (44.0)
Unfinished high school	20 (13.3)
Finished high school	36 (24.0)
University frequency	4 (2.7)
University degree	22 (14.7)
MSc or equivalent	1 (0.7)
PhD	1 (0.7)
Still actively studying, n (%)	2 (1.3)

N=153, unless otherwise noted.

^a^ Includes full-time, part-time, self-employed, and casual employment;

^b^ Includes those looking for a job and not actively looking for a job. MSc: Master of Science; PhD: Doctor of Philosophy; SD: standard deviation.

**Figure 1:**
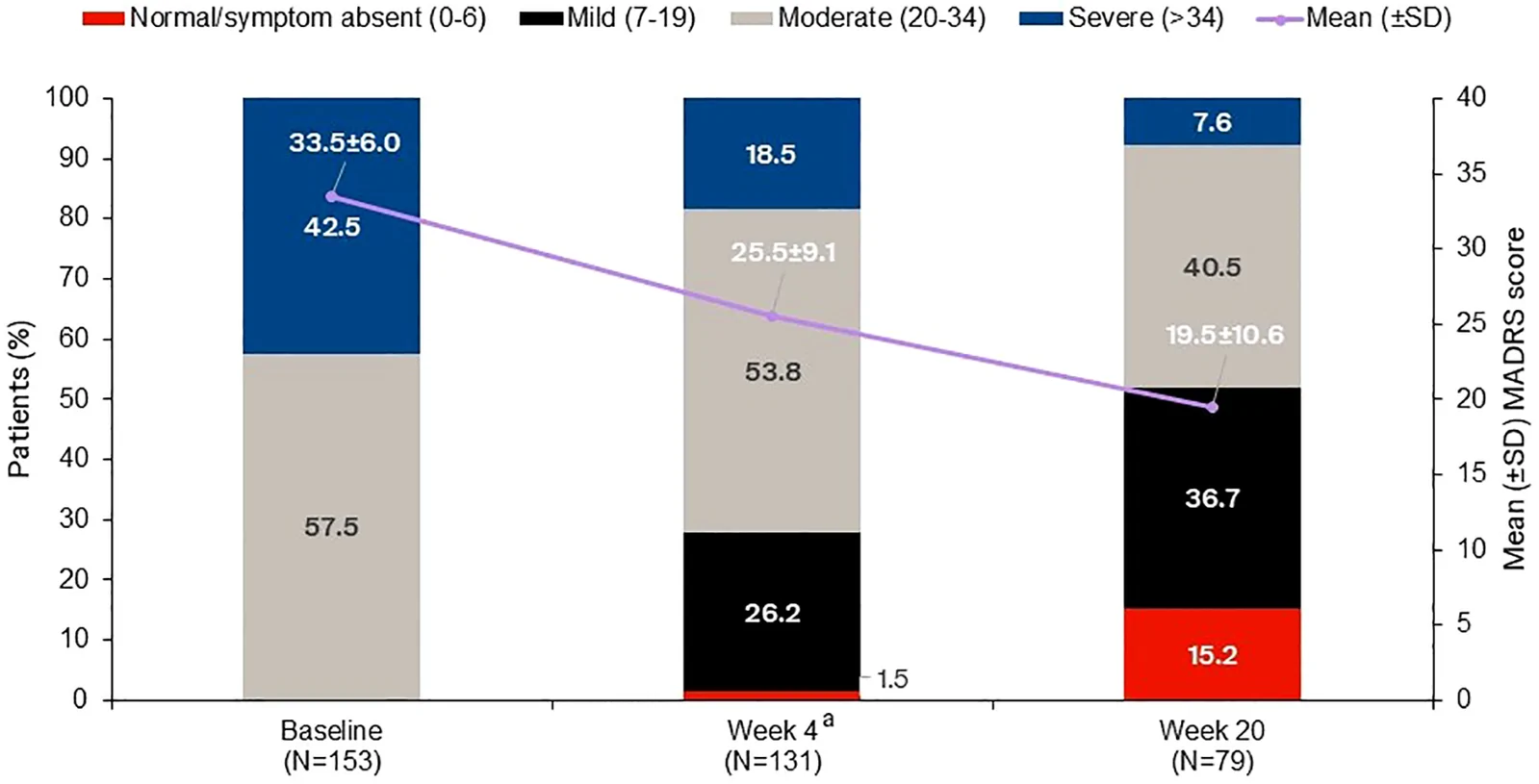
Severity shifts in MADRS score over time. FAS: N = 153. ^a^Data missing for one patient. MADRS: Montgomery-Åsberg Depression Rating Scale; FAS: full analysis set; SD: standard deviation.

**Figure 2:**
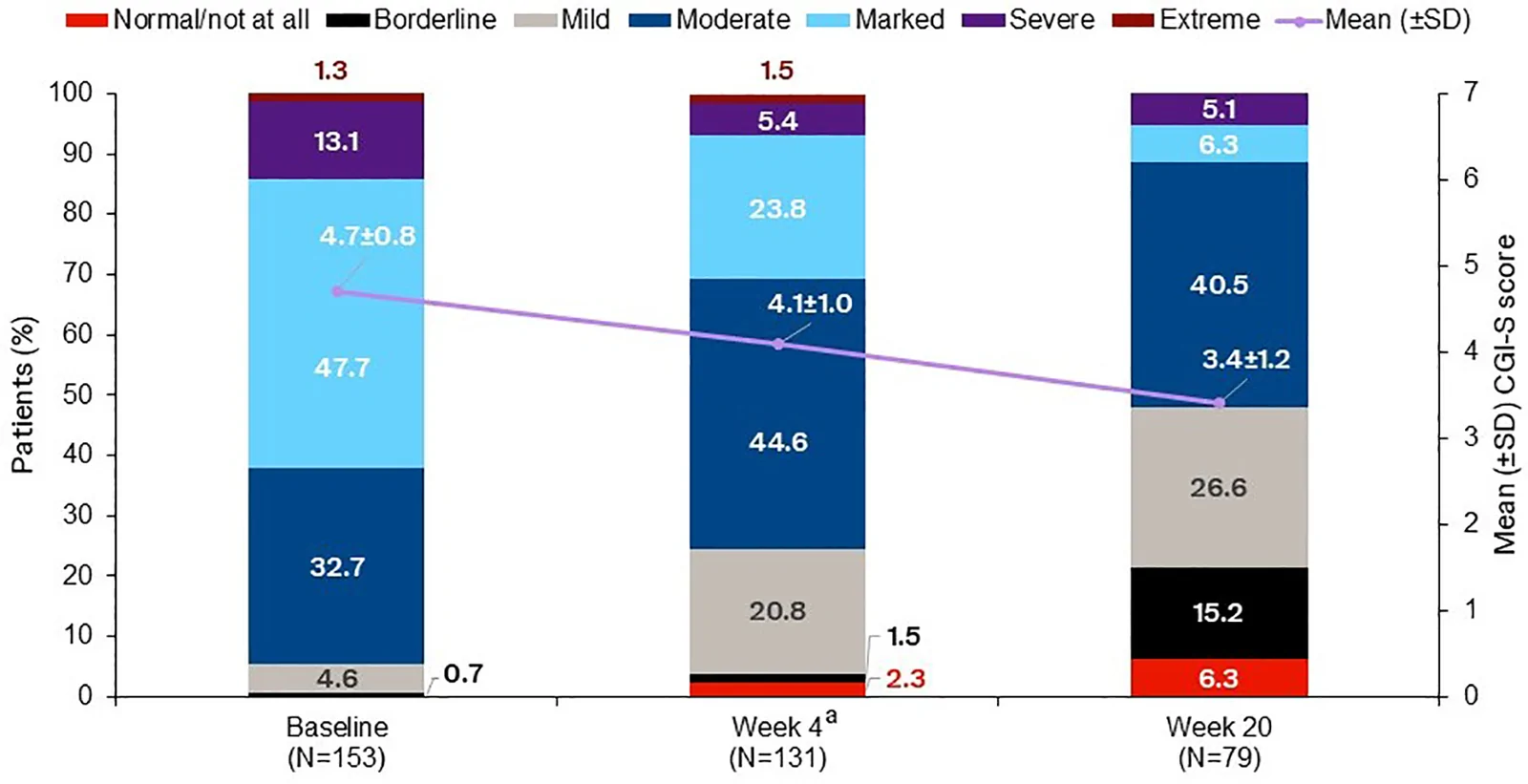
Severity shifts in CGI-S score over time. FAS: N = 153. ^a^Data missing for one patient. CGI-S: Clinical Global Impression-Severity scale; FAS: full analysis set.

All patients were classified as having moderate or severe depression, as assessed by MADRS score (moderate: 57.5%; severe: 42.5%; mean: 33.5; **Figure 1**). The mean baseline score for the CGI-S scale was 4.7, representing a ‘marked’ severity (**Figure 2**).

The first symptoms of clinical depression occurred at a mean of 38.0 years and the mean age at diagnosis of MDD was 42.0 years, with mean disease duration of 11.8 years (**Table 2**). For those with previous episodes, the mean number of episodes was 3.5 and 90.5% had recurrent major depressive episodes (**Table 2**). Most patients (98.7%) reported persistent sad, anxious, or ‘empty’ feelings, and fatigue (98.7%; **Table 3**). Many patients also presented with other depression symptoms, including feelings of hopelessness and/or pessimism (94.1%), feelings of guilt, worthlessness and/or helplessness (87.6%), and irritability/restlessness (75.2%; **Table 3**).

**Table 2:** Patient baseline clinical characteristics.

	Total (N = 153)
Age at first symptoms of clinical depression (years), mean (SD)	38.0 (14.4) (N = 151)
Age at diagnosis of MDD (years), mean (SD)	42.0 (13.7) (N = 152)
Years since diagnosis of MDD (years), mean (SD)	11.8 (11.8) (N = 152)
Duration of current MDE (months), mean (SD)	28.8 (29.9) (N = 147)
First or recurrent episode, n (%)	
First episode	47 (30.9) (N = 152)
Recurrent episode	95 (90.5) (N = 105)
Number of episodes, mean (SD)	3.5 (1.9) (N = 98)
Age at diagnosis of TRD (years), mean (SD)	52.5 (10.7) (N = 149)

MDD, major depressive disorder; MDE, major depressive episode; SD, standard deviation; TRD, treatment resistant depression.

**Table 3:** Baseline disease status.

	Total (N = 153)
Baseline disease status, n (%)	
Persistent sad, anxious, or ‘empty’ feelings	151 (98.7)
Feelings of hopelessness and/or pessimism	144 (94.1)
Feelings of guilt, worthlessness and/or helplessness	134 (87.6)
Irritability, restlessness	115 (75.2)
Loss of interest in activities or hobbies once pleasurable, including sex	151 (98.7)
Fatigue and decreased energy	151 (98.7)
Difficulty concentrating, remembering details and making decisions	145 (94.8)
Insomnia, early-morning wakefulness, or excessive sleeping	122 (79.7)
Overeating, or appetite loss	108 (70.6)
Other relevant depression-related symptoms	89 (58.2)

Overall, 74/153 (48.4%) patients had a family history of depression; 27.5% of patients were diagnosed with an additional psychiatric disorder, and comorbidities excluding psychiatric disorders were observed in 71.2% of patients (**Table 4**). Previous TRD therapy for the episode ending at or prior to baseline had been taken by 83.0% of patients, with sertraline (37/127; 29.1%), fluoxetine (36/127; 28.4%), and escitalopram (34/127; 26.8%) being the most common TRD therapies (**Table 4**).

**Table 4:** Diagnosis, medical history and previous TRD therapies at baseline.

	Total (N = 153)	
Family history of depression, n (%)	74 (48.4)	
Any other psychiatric disorder(s), n (%)	42 (27.5)	
Generalized anxiety disorder	12 (28.6)
Panic disorder	2 (4.8)
Post-traumatic stress disorder	2 (4.8)
Bulimia nervosa	2 (4.8)
Agoraphobia	1 (2.4)
Other^a^	29 (69.1)
Any other comorbidity, n (%)^b^	109 (71.2)	
Hypertension	38 (34.9)	
Dyslipidemia	35 (32.1)	
Hypothyroidism	16 (14.7)	
Asthma	11 (10.1)	
Fibromyalgia	10 (9.2)	
Type 2 diabetes mellitus	10 (9.2)	
Obesity	8 (7.3)	
Osteoarthritis	6 (5.5)	
Chronic gastritis	5 (4.6)	
Diabetes mellitus	5 (4.6)	
Hypercholesterolemia	5 (4.6)	
Migraine	5 (4.6)	
Any previous TRD therapy for the current episode ending at baseline or prior to baseline, n (%)^c^	127 (83.0)	
Sertraline	37 (29.1)	
Fluoxetine	36 (28.4)	
Escitalopram	34 (26.8)	
Venlafaxine	32 (25.2)	
Mirtazapine	20 (15.8)	
Bupropion	19 (15.0)	
Paroxetine	14 (11.0)	
TRD therapies per class, n (%)		
SSRIs	39 (30.7)	
Atypical antidepressants and SSRIs	14 (11.0)	
SNRIs and SSRIs	14 (11.0)	
Atypical antidepressants	9 (7.1)	
Atypical antidepressants and SNRIs and SSRIs	8 (6.3)	
Augmentation with antipsychotic	6 (4.7)	

^a^ Other includes: insomnia (n=21), anxiety (n=8), persistent depressive disorder (n=3), sleep disorder (n=2), impulsive behavior (n=1), somnambulism (n=1), anxiety disorder (n=1).

^b^ Only values with n ≥5 are reported;

^c^ Patients may have received more than one previous TRD therapy. Only values with n ≥5 are reported. SNRI, serotonin-norepinephrine reuptake inhibitor; SSRI, selective serotonin reuptake inhibitor; TRD, treatment resistant depression.

### Treatment characterization

**Figure 3:**
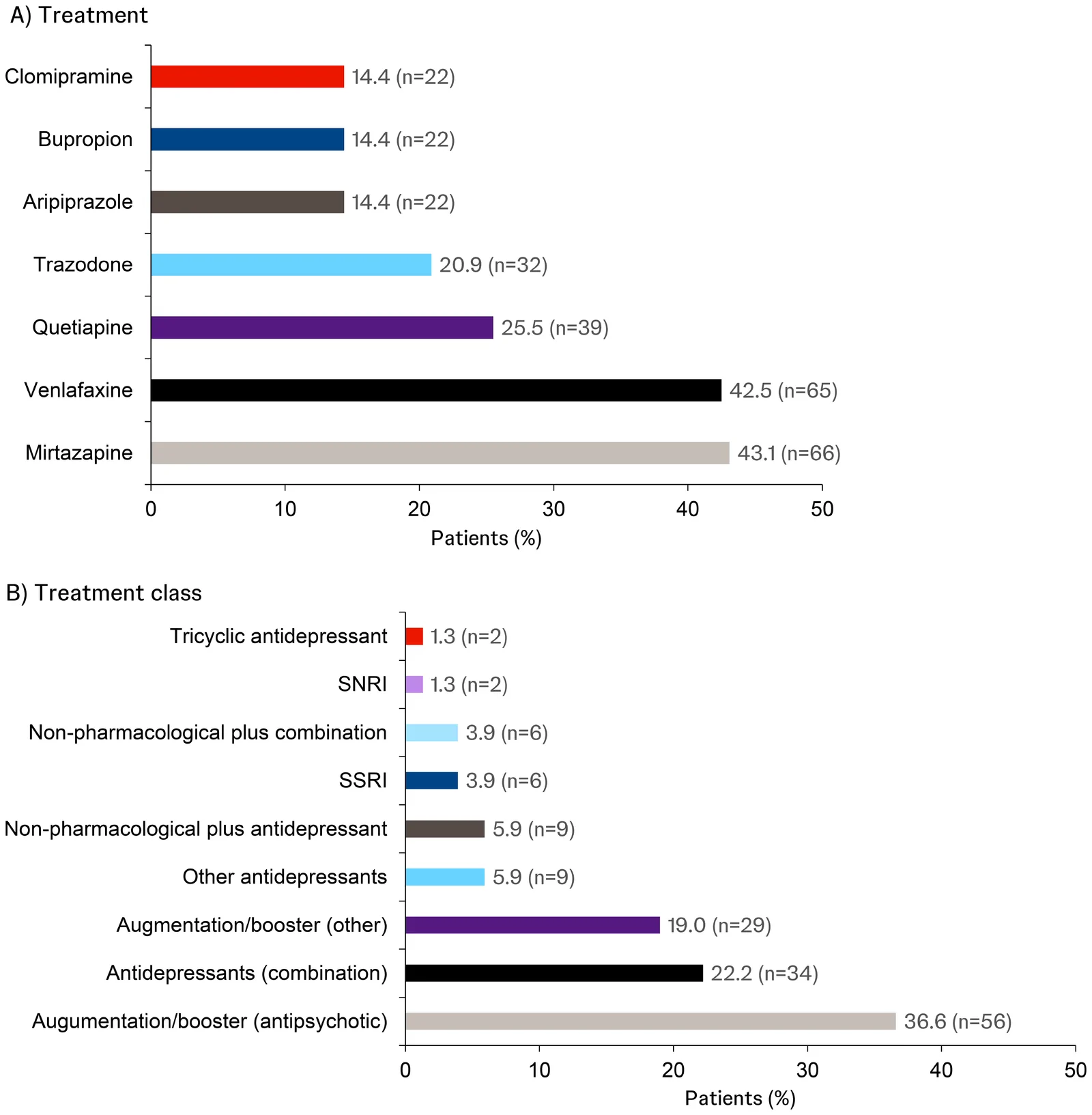
TRD therapies initiated or ongoing at baseline by **(A)** treatment and **(B)** treatment class. FAS: N = 153. The seven most common treatments are included here. FAS, full analysis set; SNRI, serotonin-norepinephrine reuptake inhibitor; SSRI, selective serotonin reuptake inhibitor; TRD, treatment resistant depression.

The most common treatments for TRD initiated or ongoing at baseline were mirtazapine (66/153; 43.1%), venlafaxine (65/153; 42.5%), quetiapine (39/153; 25.5%), and trazodone (32/153; 20.9%); **Figure 3A**). Most patients were treated with an augmentation treatment with an antipsychotic (56/153; 36.6%), combination of antidepressants (34/153; 22.2%), or other augmentation strategy (29/153; 19.0%; **Figure 3B**). More than half of the patients (80/153; 52.3%) received concomitant TRD therapies, most commonly clonazepam (20/80; 25.0%) and diazepam (19/20; 23.8%). By Week 4, 10.7% of patients (14/131) had changed TRD treatment since baseline, increasing to 11.4% (9/79) at Week 20.

### Clinical outcomes

**Figure 4:**
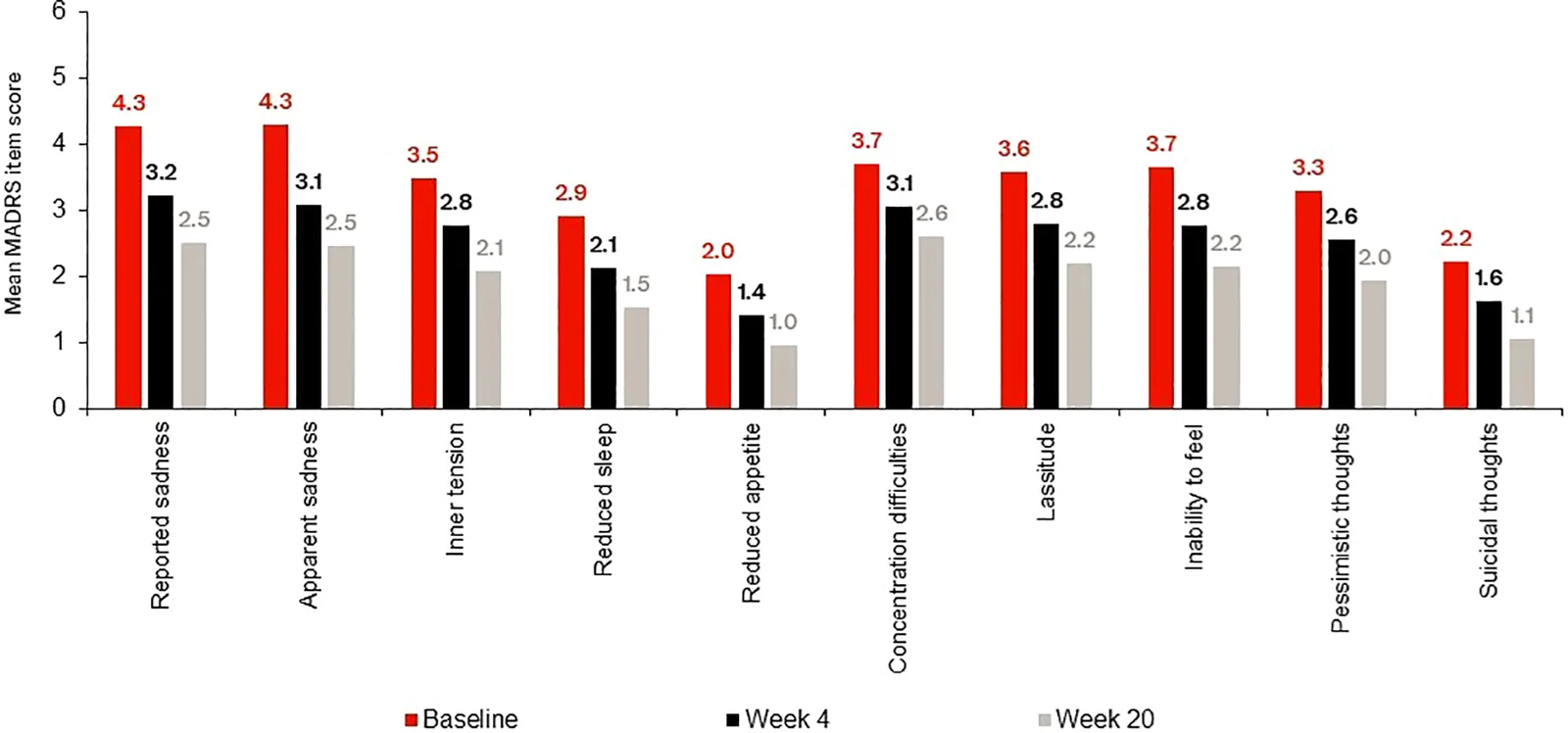
MADRS item score evolution over time. FAS; reporting individual item mean MADRS scores at baseline (N = 153), Week 4 (N = 131; Data missing for one patient), and Week 20 (N = 79). FAS, full analysis set; MADRS, Montgomery-Åsberg Depression Rating Scale.

Mean MADRS score, and corresponding disease severity, decreased over time (MADRS at Baseline: 33.5; Week 4: 25.5; Week 20: 19.5; **Figure 1**). Numerical reductions in individual MADRS item scores were also observed through Week 20 (**Figure 4**).

Clinical response and remission rates increased from Week 4 (14.9% [95% CI: 9.6%, 21.6%] response; 3.1% [95% CI: 0.8%, 7.7%] remission) to Week 20 (40.5% [95% CI: 29.6%; 52.1%] response; 25.3% [95% CI: 16.2%; 36.4%] remission). The mean percentage change in MADRS from baseline (Week 4: −23.8%; Week 20: −40.5%) was statistically significant at both timepoints (p<0.001). After GEE adjustment, only employment/occupational status was associated with percentage change in MADRS: employed patients (p=0.008) and patients dependent on their partner/spouse or students (p=0.016) demonstrated a greater reduction compared to unemployed patients.

At Week 20, patients taking SNRIs or other antidepressants at baseline showed a statistically significant regression (B=–11.146; p=0.038) and had a mean MADRS score percent change of –37.8 compared with patients taking an antipsychotic plus SNRI or other antidepressant (mean MADRS score percent change of –27.0). Employed patients (B=–18.212; p=0.003) demonstrated a statistically significant reduction in mean MADRS score percent change at Week 20 of –35.9 compared with a –19.7 change in unemployed patients.

Mean CGI-S score reduced over time (Week 4: 4.1; Week 20: 3.4). Moreover, the proportion of patients who were ‘severely ill’ or ‘among the most extremely ill patients’ reduced from baseline (13.1% severely ill; 1.3% among the most extremely ill) to Week 4 (5.4%; 1.5%) and Week 20 (5.1%; 0.0%; **Figure 2**). A statistically significant reduction from baseline in mean CGI-S score was observed at both timepoints (Week 4: −0.6, p<0.001; Week 20: −1.3, p<0.001), representing an improvement in symptoms. At Week 20, patients taking SNRIs or other antidepressants at baseline did not show a statistically significant difference in mean CGI-S score (–1.0; B=–0.198; p=0.311) compared with patients taking an antipsychotic plus SNRI or other antidepressant (mean change: –0.9).

### Patient-reported outcomes

Mean PHQ-9 scores showed statistically significant reductions from baseline (18.3) to Week 4 and Week 20 (15.1 and 12.1, respectively; mean change both p<0.001). Mean EQ-5D-5L scores increased significantly over time (baseline: 0.55; Week 4: 0.64; Week 20: 0.75; mean change both p<0.001), indicating improvements in HRQoL over time.

## Discussion

The ResisToday study aimed to gather RWE on TRD treatment in Portugal, examining the characterization, treatment patterns, and clinical and PROs of patients across a broad range of clinical practices in Portugal. These results broadly align with other studies in Portugal and Europe more widely; reductions in MADRS and CGI-S scores were seen, but clinical response and remission rates were below 50% and 30%, respectively (19, 20, 33). Previous research has shown slightly better outcomes in the context of a clinical trial, with alternative assessment measures (4). However, given the differing nature of the studies, as ResisToday was a prospective, observational study, direct comparisons with clinical trial data may not be appropriate. PRO results in this and other studies demonstrated a similar effect, with patients reporting improvements in, but not resolution of, depressive symptoms (4, 20, 34–36). These persistently poor outcomes occur despite the availability of multiple pharmacological and non-pharmacological treatments, and highlight the need for new, more effective treatments and clear treatment guidelines.

Over 80% of patients had received previous treatment for TRD, further highlighting the lack of efficacy of current treatment strategies and the protracted nature of the condition. The most commonly used therapeutics found in the current study, mirtazapine, venlafaxine, quetiapine, and trazodone, were aligned with the results from broader European studies, demonstrating the prevalence of SNRIs and antidepressants in the treatment regimen of patients with TRD (20, 22). The most common treatment strategies included augmentation/booster with an antipsychotic (36.6%) and antidepressant combinations (22.2%), of which the combination of SNRIs with ‘other’ antidepressants (11.1%) was the most common. The low levels of non-pharmacological therapy use, such as psychotherapy and ECT, are also of note and further research to understand the role of such therapies in the landscape of TRD treatment may be beneficial. However, it should be noted that alternative non-pharmacological options, such as TMS, were not available at most study sites.

The demographic profile was generally aligned with previous research, in line with Portugal’s demographics (19). However, the mean age of patients enrolled in ResisToday was 53.9 years, which is an older age profile compared with most TRD clinical trials (14, 35, 37). Characterizations of patient profiles may facilitate more informed clinical decisions and contribute to improved clinical outcomes (38). Occupational status was associated with percent change in MADRS, reporting that employed patients and patients dependent on their partner/spouse or students demonstrated a greater reduction compared to unemployed patients, potentially reflecting increased support and/or scheduling flexibility on high-symptom days. The comorbidity data in this study support those found in previous characterizations of the TRD population: compared to patients with non-TRD MDD, patients with TRD presented higher psychiatric and physical comorbidities (39, 40). For example, 71.2% of patients in this study presented with non-psychiatric comorbidities, including hypertension and hypothyroidism. Treatment type at baseline had an impact on treatment outcomes; patients taking SNRIs at baseline demonstrated a larger change in MADRS score than those taking an SNRI plus an antipsychotic, however there was no significant difference in CGI-S score. These factors highlight the increased and more complex disease burden faced by patients with TRD compared with their treatment-responsive counterparts, and the heterogeneity already observed in the clinical profiles of patients with TRD (41).

Early improvement in the first weeks of TRD treatment may serve as a useful predictor of future response. For example, previous research has demonstrated an association between partial improvement by Week 4 and subsequent remission at Week 6; moreover, although treatment switching after 4 weeks for patients demonstrating no improvements was shown to have a modest benefit, an earlier switch (i.e. at Week 2) was associated with reduced rates of remission and quality of life (42). This demonstrates the potential clinical importance of the Week 4 timepoint as a key stage for clinicians to evaluate patients’ response to treatments, in alignment with the primary endpoints used across multiple studies (24, 43–46). In the present report, inclusion of a 20-week follow-up is valuable given that patients with TRD demonstrate high recurrence rates, with many patients relapsing within one year of remission (47). Therefore, it is important to evaluate patient outcomes beyond the acute phase, assessing maintenance of response over the longer term to provide insights into patient prognosis with different treatment strategies.

The low rates of response and remission at the 20-week follow-up period may be attributable to several factors. For example, as previously discussed, a substantial proportion of patients presented with comorbidities, which may have contributed to poor outcomes. Previous research in MDD has demonstrated that patients with physical or psychiatric comorbidities experience significantly worse outcomes with antidepressant treatments compared to those without comorbidities (48). Moreover, the mean duration since patients’ diagnosis of MDD was 11.8 years, with a mean gap of 4.0 years between the onset of clinical depression symptoms and diagnosis. These baseline characteristics highlight the chronic nature of TRD, compounded by diagnostic delays. Such delays may contribute to poor prognosis for patients, as valuable opportunities to initiate treatments during the early stages of symptom presentation may be missed. As such, these findings underscore the need for improvements in clinical pathways that facilitate early identification of TRD and timely initiation of efficacious intervention for patients presenting with symptoms of clinical depression.

Barriers specific to Portuguese patients must also be recognized. Portugal is a heterogeneous country when it comes to healthcare, with the national healthcare system having limited resources (19). For example, even in hospitals that have ECT as an available treatment, access is limited due to resources (such as requiring an anesthesiologist) and long waiting lists; even fewer hospitals offer treatments such as TMS (49). There is more availability of these treatments in the private sector, however availability is still considered low due to lack of economic benefit in treating psychiatric disorders.

These results broadly reflect those seen in the Portuguese real-world clinical setting, allowing for a better overview of treatment options and their specific effectiveness, compared with previous studies looking at patients with TRD across multiple countries, while also highlighting the need for better resource planning and management (20). Given that most patients did not reach response or remission during this 20-week study, as seen across TRD research, the full range of pharmacological and non-pharmacological treatment strategies should be considered when treating patients with TRD. TRD is hypothesized as biologically distinct from MDD in newer perspectives, with distinct neurobiological functions, particularly reduced glymphatic drainage clearance (50). This can lead to accumulation of toxins and raised cortisol levels, which provide a target for pharmacological mechanisms as well as non-pharmacological mechanisms such as psychotherapy and ECT as well as the use of deep sleep and circadian alignment (3, 19, 50, 51). Additionally, although psychotherapy and ECT are available in the Portuguese Serviço Nacional de Saúde (National Health Service), they were not commonly used in this study. These and other approved treatments, such as repetitive TMS and glutamatergic agents/modulators, such as esketamine, can substantially broaden the TRD treatment arsenal and produce faster results than more commonly used SSRIs and SNRIs (14, 37, 52–56).

### Strengths and limitations

A key strength of this study lies in the heterogeneity of the sample population, which enhances generalizability of these findings across diverse clinical contexts. Moreover, the diversity of sites and the inclusion of widely available and utilized therapeutics enhance the overall generalizability of these results.

The RWE collected here represents Portuguese Serviço Nacional de Saúde (National Health Service) clinical practice. However, considering the growing importance of private healthcare services in Portugal, this study may not fully represent the reality in Portugal, and countries in which the private healthcare sector is more prominent should avoid comparisons. Similarly, although results are comparable to other countries that have a broad arsenal of therapeutics available in the public health service, generalizability to countries and health systems with more limited or stricter access to therapeutics, or a higher proportion of rural areas, may be limited.

Although the present study reports real-world evidence across a broad spectrum of antidepressant treatments, patients with various psychiatric comorbidities, such as personality disorders, were excluded (**Supplementary Table 1**). Consequently, the findings of this analysis are applicable only to patients with a singular diagnosis of TRD and comparisons with the broader patient population living with multiple psychiatric conditions should be approached with caution. Given the high rates of co-occurrence of personality disorders within the TRD population, future research assessing clinical outcomes for multimorbid patients may be beneficial (57).

A notable limitation of the present analysis is the high overall rate of attrition, with 44.4% of patients discontinuing during the study period. While the sample size was adequately powered to identify clinically meaningful differences, the reduction in sample size performed in accordance with information collected from the participating sites led to a higher margin of error in the inferences performed. The large proportion of patients having discontinued study involvement by Week 20 introduces the possibility of survivor bias, which must be considered when interpreting the results of this study. Patients who responded poorly to treatment may have been more likely to discontinue and therefore may not be fully represented in reported clinical outcomes; to address this limitation, reasons for discontinuation are also reported, providing reassurance that lack of efficacy was not the reason that led to discontinuation in the majority of cases. Additionally, as is typical in observational studies, some variables were more difficult to control which may impact the results of this study. Examples of this include disease severity and time from diagnosis, which were not considered as covariates in the GEE analysis. A longer follow-up period and larger sample size would also allow for better and more robust inferences on the different patient profiles and the corresponding treatment regimens.

A limitation of this study was that although clinical response and remission rates are reported, maintenance of each outcome and analyses that could describe the magnitude of improvements (e.g. Cohen’s d) were not assessed. Further analyses to assess the stability of improved outcomes would provide greater granularity for clinicians to better understand outcomes for patients with TRD in Portugal.

Future research should focus on healthcare resource utilization, especially given that resource scarcity and budget constraints are becoming ubiquitous in high-income countries. Additionally, subgroup analysis considering responders versus non-responders, analysis by treatment class, and analysis across occupational subsets may provide clinically important insights.

## Conclusions

The results of ResisToday highlight a known reality in TRD, where current standard of care does not achieve the desired clinical and patient-reported outcomes, regardless of treatment regimen. Diversity and lack of consensus on treatment strategies is seen in Portugal and across Europe (20). This issue must be addressed to develop improved treatment protocols that can deliver effective solutions for patients with TRD in Portugal.

## Data Availability

Anonymized patient group data that support the conclusions of this article will be made available by the authors, without undue reservation.

## Supplementary material

Supplementary Table 1 Inclusion and exclusion criteria.

Supplementary Figure 1 Patient disposition. AE: adverse event; FAS: full analysis set; TRD: treatment resistant depression.
