# Supplementary material for: Patient characteristics, treatment patterns and clinical response in patients with treatment resistant depression starting a new treatment in the clinical setting: a multicenter, observational study in Portugal
Source: Front Psychiatry. 2026 Aug 13;17:1749006. doi: 10.3389/fpsyt.2026.1749006 (PMC13520672; doi:10.3389/fpsyt.2026.1749006)
Supplement: Supplementary file 1 [file SupplementaryFile1.pdf]

SUPPLEMENTARY APPENDIX

Supplementary Table S1. Inclusion and exclusion criteria

Inclusion criteria	Exclusion criteria
1. Adult male or female (≥ 18 years old)	1. Inability to communicate orally
2. Met the diagnostic criteria for single episode or recurrent MDD, without psychotic features, according to either the Tenth Revision of the International Statistical Classification of Diseases and Related Health Problems (ICD-10) or the Diagnostic and Statistical Manual of Mental Disorders, Fifth Edition (DSM-5)	2. Institutionalized patients
3. Suffered from a moderate or severe depressive syndrome, as defined by a Montgomery-Åsberg Depression Rating Scale (MADRS) total score ≥22 at baseline	3. Current or prior DSM-5 diagnosis of a psychotic disorder or MDD with psychotic features, bipolar or related disorders (according to the investigator's clinical judgment and/or confirmed by the Mini International Neuropsychiatric Interview [MINI]), obsessive compulsive disorder (current only), intellectual disability (DSM-5 diagnostic codes 317, 318.0, 318.1, 318.2, 315.8, and 319), autism spectrum disorder, borderline personality disorder, antisocial personality disorder, histrionic personality disorder, or narcissistic personality disorder
4. Met the treatment resistant depression (TRD) criteria, defined as lack of clinically meaningful improvement, with at least 2 different antidepressant treatments in the current episode of depression, prescribed in adequate dosages for adequate duration (at least 6 weeks) with adequate treatment adherence, assessed by the Massachusetts General Hospital – Antidepressant Treatment Response Questionnaire (MGH-ATRQ)	4. Homicidal ideation/intent or suicidal ideation with some intent to act within 6 months prior to the start of the study per the investigator's clinical judgment and/or based on the Columbia-Suicide Severity Rating Scale (C-SSRS)

5. Initiated a new antidepressant treatment to treat the current depressive episode	5. History of moderate or severe substance or alcohol use disorder (except for nicotine and caffeine) according to DSM-5
6. Informed consent form signed by the patient	6. Lifetime history of hallucinogen-related substance use disorder, with ketamine, phencyclidine (PCP), lysergic acid diethylamide (LSD), or 3,4-methylenedioxy-methamphetamine (MDMA)
7. Must have been able to read, understand and complete PROs	7. Enrolled in any clinical trial or observational study at study enrollment
	8. Had previously received esketamine or ketamine during the ongoing episode or were about to receive it as a new antidepressant treatment

C-SSRS: Columbia-Suicide Severity Rating Scale; DSM-5: Diagnostic and Statistical Manual of Mental Disorders, Fifth Edition; ICD-10: Tenth Revision of the International Statistical Classification of Diseases and Related Health Problems; LSD: lysergic acid diethylamide; MADRS: Montgomery-Åsberg Depression Rating Scale; MDD: major depressive disorder; MDMA: 3,4-methylenedioxy-methamphetamine; MGH-ATRQ: Massachusetts General Hospital – Antidepressant Treatment Response Questionnaire; MINI: Mini International Neuropsychiatric Interview; PCP: phencyclidine; PRO: patient-reported outcome; TRD: treatment resistant depression.

Supplementary Figure S1. Patient disposition

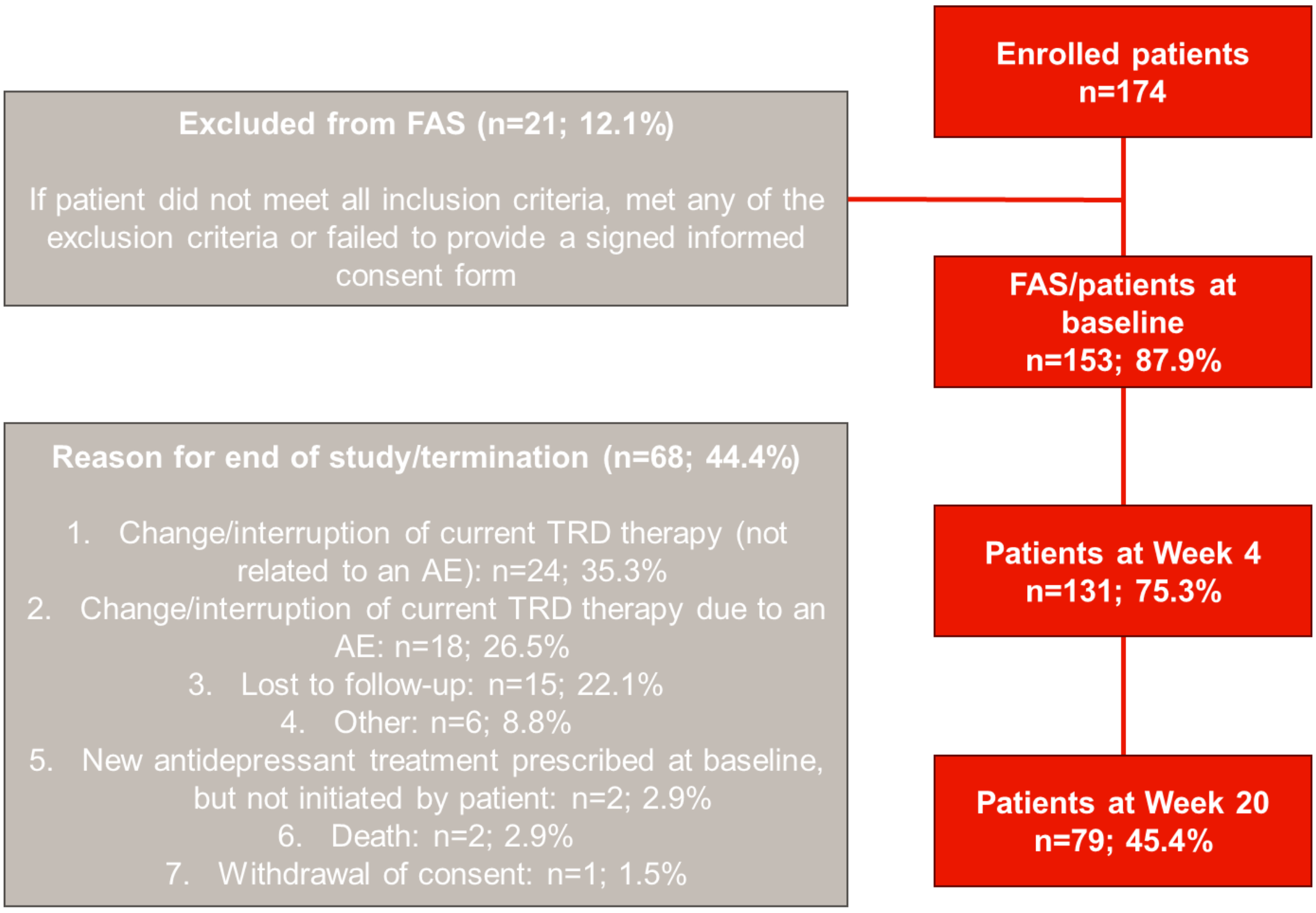

AE: adverse event; FAS: full analysis set; TRD: treatment resistant depression.
